# Exploring Knowledge and Perception of Artificial Intelligence in Teaching Dental Hospitals of Peshawar: A Cross-Sectional Study

**DOI:** 10.7759/cureus.97935

**Published:** 2025-11-27

**Authors:** Sumbal Iftikhar, Ahmad Deedar Khan, Sakina Anjum, Muhammad Awais, Muhammad Aqeel Ajmal, Aima Fatima Mirza, Zar Khan, Saira Afridi

**Affiliations:** 1 Dental Surgery, Sardar Begum Dental College, Peshawar, PAK; 2 Dental Surgery, Fatima Jinnah Dental College, Karachi, PAK; 3 Preventive Medicine, Sardar Begum Dental College, Peshawar, PAK

**Keywords:** artificial intelligence, dental health, dentistry, knowledge, perceptions

## Abstract

Background: Artificial intelligence (AI) has become an essential tool in all fields, including dentistry. Different levels of familiarity with the knowledge regarding AI and perception among dental professionals exist globally, with many advocating for its integration into the curriculum. However, highlighting ethical concerns like data privacy, biases, and job displacement must be addressed to ensure its responsible implementation in dentistry.

Objective: This study aimed to assess the knowledge and perception of AI in dentistry among dentists working in teaching dental hospitals of Peshawar.

Methods: This analytical cross-sectional study was conducted from January 2024 to June 2024 at Sardar Begum Dental College and Hospital (SBDC). The population included 475 total dentists, including house officers, postgraduate trainees, and faculty members, selected through a census sampling technique. Participants were required to have a valid Pakistan Medical & Dental Council (PMDC) registration and willingness to participate, while those unwilling or unavailable were excluded.

Results: Among the 475 participants, 49.3% had low knowledge, 48% moderate, and 2.7% high. Faculty members demonstrated greater knowledge compared to house officers and trainees. Most participants agreed that AI enhances efficiency and quality in dentistry and should be incorporated into the curriculum. Ethical and data privacy concerns were more pronounced among younger participants. Interest in learning more about AI was high, particularly among house officers. Perceptions varied significantly by designation and age, but not by gender.

Conclusion: Although knowledge of AI in dentistry remains limited, attitudes toward its integration are largely positive. Bridging knowledge gaps and addressing ethical challenges are essential for the effective and responsible incorporation of AI into dental education and clinical practice.

## Introduction

Artificial intelligence (AI), once a nebulous concept, has become a ubiquitous tool across all aspects of life in the last two decades. It is a widely debated subject, and its integration into our daily lives has grown rapidly. AI can be described as the ability of a device to carry out tasks without the need for human cognitive abilities, a feat previously thought to be unattainable before the 1950s [1].

The contemporary workforce emphasizes the need to keep our options open and continuously strive to stay ahead in our careers. It is essential to remain abreast of new technological advancements to ensure professional progress [2,3].

The integration of AI across various medical domains has significantly impacted diagnostics, particularly in imaging and radiography, where it plays a pivotal role. AI facilitates clinical decision-making, assists in treatment planning, and predicts treatment outcomes, thereby reducing the workload and time required for procedures while maintaining precision [4]. This efficiency positions AI as an indispensable asset in the global healthcare landscape [5,6].

In dentistry, AI serves as a critical tool across specialties [7], encompassing applications in diagnosis, pre-operative treatment planning, patient documentation, outcome assessment, and follow-up care. Its versatility underscores its significance in modern dental practice, establishing it as an essential component of the field [8,9].

AI's capabilities in dentistry range from the detection of dental caries, vertical root fractures, apical lesions, and volumetric assessment of pulp space to the evaluation of tooth wear [10,11]. Studies have shown that AI can diagnose lesions more efficiently and accurately compared to human dentists.

Furthermore, AI is utilized for the detection of anatomic landmarks through cephalogram, cone beam computerized tomography (CBCT), and magnetic resonance imaging (MRI) scans [12,13], as well as in the design of complete and partial dentures, crowns, and bridges. Its ability to assess pocket depths, identify bone loss, and classify conditions such as oral cancer, gingivitis, and periodontitis underscores its importance in dental practice.

Research conducted in various countries, including Saudi Arabia [14], India [15], and South America [16], indicates varying levels of awareness and perception of AI in dentistry among dental professionals. Another study in Tamil Nadu, India, showed a 92.39% majority in favor of adding AI to the curriculum, while 35.87% opposed the idea due to their insecurities [17]. According to recent research, in Karachi, 17% of house officers and dental students, 22% of postgraduates, and 18% of general dentists demonstrated awareness of AI-driven applications. Conversely, in Peshawar, a notable 61.8% of dental students exhibited awareness of AI [18]. The majority of dentists in these studies recognized the potential for AI to advance the field, although concerns regarding ethical issues, such as biases, data privacy, security, surveillance, and potential job displacement, were also noted. These findings highlight the importance of addressing ethical considerations in the integration of AI in dentistry.

Therefore, this study aimed to assess and compare the knowledge and perception of AI in dentistry among dentists working in teaching dental hospitals of Peshawar. The study specifically examined differences across age groups, gender, and professional designation, with knowledge considered the primary outcome and perception as a secondary outcome. Peshawar was selected because of its diverse representation of public and private teaching dental institutions, which collectively reflect a broad cross-section of the region’s dental professionals.

The thresholds for knowledge levels (low: ≤3, moderate: 4-6, high: ≥7 correct responses) were adopted based on previous surveys assessing AI knowledge among dental professionals [14,15]. This framework allows for consistent comparison with prior studies.

## Materials and methods

The study was designed as an analytical cross-sectional study and was conducted at Sardar Begum Dental College and Hospital (SBDC) in Peshawar, Pakistan, over a duration of six months from January 2024 to June 2024. The study population comprised house officers, postgraduate trainees, and faculty members. A total of 255 house officers, 146 postgraduate trainees, and 74 faculty members participated in the study. Census sampling was used to include all eligible dentists in SBDC, a private dental teaching institution in Peshawar. This approach was feasible due to the limited total population size and ensured comprehensive participation. Of the 475 eligible dentists, all consented to participate; no refusals or dropouts were recorded. The inclusion criteria consisted of all house officers, postgraduate trainees, and faculty members of teaching dental colleges and hospitals in Peshawar who held valid Pakistan Medical & Dental Council (PMDC) registration licenses and were willing to participate. The exclusion criteria included individuals who were not willing to participate or were unavailable at the time of data collection. As a census sampling technique was employed, no formal sample size calculation was performed. However, the total sample size of 475 participants was adequate, as similar studies assessing AI knowledge among dental professionals reported comparable or smaller sample sizes [14,15].

The questionnaire items were adapted from previously validated tools assessing AI knowledge and perception in dentistry [14-17]. It consisted of 21 items: nine for knowledge and 12 for perception. Knowledge items were scored as correct (1) or incorrect (0). Participants were classified as having low (≤3 correct), moderate (4-6 correct), or high (≥7 correct) knowledge, following established thresholds from prior studies [14,15]. A sample of the questionnaire is available in the Appendix for replication purposes. Content validity was established through expert review by three senior faculty members. The questionnaire was pretested in a pilot study with 20 participants to ensure clarity, and modifications were made accordingly. Reliability testing yielded a Cronbach’s alpha of 0.82, indicating good internal consistency.

Ethical approval for the study was obtained from the Gandhara University Ethical Committee for Research (Ref. No: GU/2024/156). Written informed consent was obtained from all participants prior to data collection, and confidentiality of the information provided was strictly maintained.

Data were analyzed using IBM SPSS Statistics for Windows, Version 26.0 (released 2018, IBM Corp., Armonk, NY). Descriptive statistics were calculated as frequencies and percentages. Associations between knowledge/perception levels and demographic variables (age, gender, designation) were tested using chi-square analysis. A p-value < 0.05 was considered statistically significant.

## Results

This study aimed to evaluate the knowledge and perception of dentists associated with various teaching hospitals in Peshawar regarding the role of AI in dentistry. A total of 475 participants who met the inclusion criteria were evaluated.

Table 1 represents the demographic traits of the study participants. Of the participants, 239 (50.3%) were men, and 236 (49.7%) were women. The participants were divided into five age groups: 20-25, 26-30, 31-35, 36-40, and 41 and above. This categorization was done to analyze age-related differences within the dataset. Of the respondents, 255 (53.7%) were house officers, 146 (30.7%) were training medical officers, and 74 (15.6%) were members of the faculty.

**Table 1:** Demographic traits

Variable	Frequency (n)	Percentage (%)
Age		
20-25 years	206	43.4
26-30 years	189	39.8
31-35 years	45	9.5
36-40 years	13	2.7
41 years and above	22	4.6
Gender		
Male	239	50.3
Female	236	49.7
Designation		
House officer	255	53.7
Training medical officers	146	30.7
Faculty	74	15.6

Table 2 presents the knowledge of the respondents regarding the role of AI in dentistry. Participants were divided into three groups based on their level of knowledge and evaluated by their performance on a set of nine questions pertaining to the role of AI in dentistry. Participants who answered three or fewer questions correctly were categorized as having a low level of knowledge. Participants who answered between four and six questions correctly were categorized as having a moderate level of knowledge. Those who answered seven or more questions correctly were categorized as having a high level of knowledge.

**Table 2:** Level of knowledge regarding artificial intelligence (AI) in dentistry

Classification	Frequency (n)	Percentage (%)
Low level of knowledge	234	49.3%
Moderate level of knowledge	228	48%
High level of knowledge	13	2.7%

Of the total participants, 234 (49.3%) had a low level of knowledge, 228 (48%) had a moderate level of knowledge, and 13 (2.7%) had a high level of knowledge regarding the role of AI in dentistry.

The perception of the participants regarding the role of AI in dentistry was assessed through a series of statements using a Likert scale. Table 3 presents the categorization of responses across five levels of agreement for each statement. A majority of participants (88%) agreed that the usage of AI in dentistry is beneficial. Similarly, 86.7% believed that AI can improve the efficiency of dentists. 85.7% of respondents agreed that AI can improve the quality of dental work. 75.8% felt that AI is capable of bringing forth innovative solutions in dentistry. Opinions on the affordability and accessibility of AI for dentists were varied, with a similar number of respondents having differing opinions regarding these statements. A majority of the respondents (80.4%) agreed with the usage of AI in dental practice as well as its incorporation into the dental curriculum (66.7%). A majority of the respondents were not concerned about AI replacing dentists permanently (56%). Ethical concerns were noted by 59.2% of the respondents, and data protection and privacy issues by 68.8%. A substantial number of participants (80.7%) expressed interest in exploring more about AI in dentistry.

**Table 3:** Perception regarding artificial intelligence (AI) in dentistry

Serial No.	Perception regarding AI	Strongly agree	Agree	Neutral	Disagree	Strongly disagree
1	Artificial intelligence use in dentistry is beneficial.	257 (54%)	158 (33%)	31 (7%)	8(2%)	21 (4%)
2	Artificial intelligence can improve the efficacy of dentists.	38.1%	48.6%	8.0%	4.4%	0.8%
3	Artificial intelligence can improve the quality of dental work in dental practice.	41.7%	44.0%	9.7%	2.7%	1.9%
4	Artificial intelligence is capable of bringing forth Innovative solutions in dentistry.	40.4%	35.4%	16.0%	5.3%	2.9%
5	Artificial intelligence software that is used in dentistry is affordable.	13.1%	20.6%	30.5%	31.2%	4.6%
6	Artificial intelligence is easily accessible to dentists and patients.	16.4%	27.4%	23.4%	27.4%	5.5%
7	Artificial intelligence should be used in dental practice.	34.5%	45.9%	9.1%	5.7%	4.8%
8	Artificial intelligence should be incorporated in the dental curriculum.	32.2%	34.5%	12.2%	10.5%	10.5%
9	Artificial intelligence may replace dentists permanently.	8.0%	21.1%	14.9%	38.3%	17.7%
10	The use of artificial intelligence may have ethical concerns.	23.4%	35.8%	30.7%	6.5%	3.6%
11	The data protection and privacy issues associated with AI use can be a concern.	26.3%	42.5%	24.0%	3.6%	3.6%
12	I am interested in exploring more about artificial intelligence in dentistry.	49.1%	31.6%	11.4%	3.2%	4.8%

Table 4 shows the association of the knowledge level of the participants with their designation. A significant difference was found among the three different designations evaluated (p = 0.008). A significant portion of the training medical officers (52.7%) and house officers (52.5%) had a low level of knowledge regarding the role of AI in dentistry. Faculty members ranked higher, with 63.5% having a moderate level of knowledge.

**Table 4:** Association of knowledge with the designation of the participants Chi-square test applied

Designation	Low level of knowledge		Moderate level of knowledge	High level of knowledge	P-value
House officers	134 (57.2 %)	117 (51.3%)		4 (30.77%)	0.008
Training medical officers	77 (32.9%)	64 (28.0 %)		5 (38.46%)	
Faculty	23 (9.8%)	47 (20.6%)		4 (30.77%)	
Total	234	228		13	

Table 5 presents the association of the level of knowledge with the participants' age. A significant difference was found in the level of knowledge (p = 0.001), with a significant portion of the participants in the 20-25 age range having a low level of knowledge (53.4%). The 41 and above age group ranked highest, with only 31.8% characterized as having a low level of knowledge.

**Table 5:** Association of knowledge with the age of the participants Chi-square test applied

Age	Low level of knowledge	Moderate level of knowledge	High level of knowledge	P-value
20-25 years	110 (47.01%)	92 (40.3%)	4 (30.7)	.001
26-30 years	92 (39.32%)	92 (40.3%)	5 (38.4%)	
31-35 years	19 (8.12%)	26 (11.4%)	0	
36-40 years	6 (2.56%)	7 (3.0%)	0	
41 and above	7 (2.99%)	11 (4.8%)	4 (30.7%)	
Total	234	228	13	

Table 6 presents the association of the gender of participants with their level of knowledge regarding the role of AI in dentistry. A significant association was found (p < 0.001), with 53% of women having a moderate level of knowledge compared to 51.5% of men having a poor level of knowledge regarding the role of AI in dentistry.

**Table 6:** Association of knowledge with the gender of the participants Chi-square test applied

Gender	Low level of knowledge		Moderate level of knowledge		High level of knowledge		P-value
Male	123 (52.5%)		103 (45.1%)		13 (100%)		< .0001
Female	111 (47.4%)		125 (54.8%)		0		
Total	234		228		13		

The perception of the respondents regarding the role of AI in dentistry was statistically correlated with their designation, age, and gender.

Compared with designation, a significant difference was found in the perception regarding the use of AI being beneficial in dentistry (p <0.001). Most house officers were in agreement, while the responses from the faculty were more distributed. There was a significant difference in the interest to learn more about the use of AI in dentistry (p 0.002), with house officers being keener than the training medical officers and the faculty.

Compared with age, a significant difference was found regarding the ethical concerns over the use of AI (p < 0.001). The greater concern was seen in the younger age groups. Similarly, the younger age groups were more concerned about data protection and privacy issues compared to the older age group (p = 0.002).

No statistically significant difference was found relating to the gender of the participants.

## Discussion

AI applications in healthcare are advancing quickly and becoming increasingly popular in both academic and clinical settings. This technology has the potential to improve learning and create large-scale decision support systems, which could significantly enhance the efficiency of healthcare delivery [5]. Currently, AI is being utilized for diagnosis, prognosis, treatment optimization, and predicting outcomes. The real-time insights and predictions generated by these systems play a vital role in minimizing the therapeutic and diagnostic errors often associated with manual processes [4].

The field of dentistry is also embracing the rise of AI. Various AI tools are now being implemented in multiple facets of dental practice, including diagnosis, treatment planning, and follow-up monitoring [7-9]. In light of this trend, a study was conducted to assess the knowledge and perceptions of dentists regarding AI in Peshawar.

The results showed that only 2.7% of dentists reported having a high level of knowledge about AI in dentistry. By contrast, 48% had moderate knowledge, while 49.3% were classified as having low knowledge. Faculty members tended to have better awareness compared to house officers and training medical officers, indicating that clinical experience and academic discussion could contribute to greater understanding. When we compare these findings to other regions, they reveal similar or slightly lower awareness levels. For example, in Saudi Arabia, about 49.4% of participants were familiar with AI, and 44.5% had a basic grasp of its principles [14]. In Turkey, 48.4% held basic knowledge about AI, whereas 78.9% had at least heard about it [19]. In stark contrast, a study in Delhi, India, found that 82.5% of dentists had some level of knowledge regarding AI [15].

The relatively lower awareness among dentists in our study may be due to limited exposure in curricula, fewer continuing education programs focused on AI, and the slower adoption of digital technologies in local teaching hospitals. Indian dental institutions have, in contrast, incorporated AI topics more thoroughly into their educational programs, which likely accounts for their higher awareness rates [14,15].

In a previous study conducted in Karachi, it was found that only 17-22% of respondents across various professional levels were aware of AI-driven applications in dentistry. Interestingly, 61.8% of dental students in Peshawar had heard of AI, indicating that awareness is indeed growing, but structured training and practical application are still in short supply [18]. This gap leads to a more superficial understanding of AI concepts rather than a comprehensive one. Despite the gaps in knowledge, our findings indicated an overall positive perception of AI among dental professionals. Most respondents acknowledged that AI has the potential to improve efficiency, quality of care, and innovation in dental practice. These findings align with international trends, where dentists tend to express optimism about AI's role in the field. For example, in South America, 86% of dentists believed that AI could lead to significant advancements [16]. Similarly, a study in Tamil Nadu, India, showed that 92.39% supported incorporating AI into dental curricula, although 35.87% were concerned about job security [17]. This mirrors our results, where enthusiasm for AI integration coexists with worries about its impact on the dental workforce.

Notably, 44% of our participants expressed fears that AI could replace human practitioners, a sentiment echoed by respondents from Saudi Arabia (40.3%), Turkey (28.6%), and Kenya (64.2%) [20]. This shared apprehension across different regions underscores a global uncertainty regarding the ethical and professional implications of AI, highlighting the need to frame AI as a supportive technology rather than a substitute for clinical expertise. These findings suggest that future training programs should emphasize the complementary role of AI alongside clinical judgment to mitigate professional apprehensions.

The general positivity surrounding AI could be a reflection of increased exposure through digital imaging systems, diagnostic tools, and social media. However, differing opinions on affordability and accessibility indicate that infrastructural and economic challenges are still hindering AI adoption, especially in developing contexts like Pakistan.

While the study identified significant associations between knowledge levels and demographic variables such as age and designation, no significant gender-based differences were observed. This finding supports the hypothesis that awareness disparities are more closely related to professional experience than to gender. Non-significant results were reviewed and are included in the overall interpretation to ensure balanced reporting. These outcomes align with the study’s objective of evaluating both knowledge and perception patterns among diverse professional subgroups.

Limitations

As a single-city study limited to teaching hospitals in Peshawar, the findings cannot be generalized to all regions of Pakistan. The cross-sectional design captures associations but not causality. Data were self-reported, which introduces potential recall and social desirability bias. Despite adapting items from validated sources, measurement bias may persist due to contextual differences, and the arbitrary thresholds for categorizing knowledge levels could have influenced classification accuracy. Furthermore, the absence of the full questionnaire in the published article may limit external reproducibility, though it is available upon request. Confounding variables such as prior exposure to digital tools or institutional differences were not controlled for and may have affected the results.

Future multicenter and longitudinal studies are recommended to validate these findings and to explore the impact of AI-related education interventions. Studies incorporating objective knowledge assessments and larger, more diverse samples would further strengthen evidence on AI awareness among dental professionals.

## Conclusions

Dentists in Peshawar demonstrate positive perceptions of AI in dentistry but exhibit varied levels of knowledge, highlighting a clear educational gap. Although the benefits of AI are widely recognized, ethical and data privacy concerns persist, particularly among younger professionals. Within the limits of a single-center cross-sectional study, these results suggest that integrating AI-related content into dental curricula may be warranted, but broader multicenter and longitudinal investigations are necessary before definitive educational policy recommendations can be made.

## Figures and Tables

**Figure 1:**
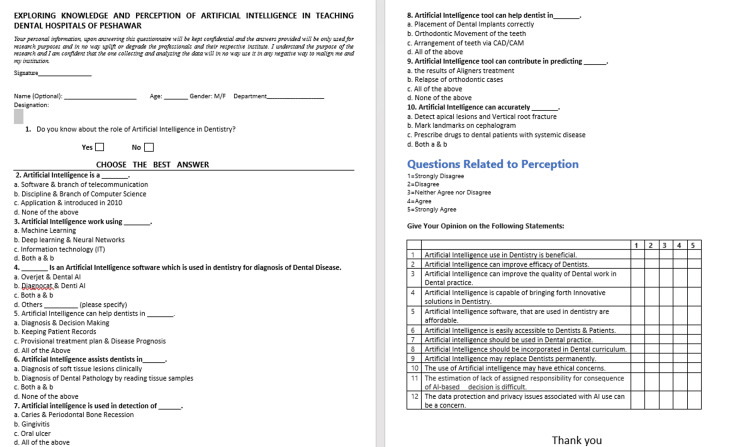
Questionnaire
